# Supplementary material for: CLN3 transcript complexity revealed by long-read RNA sequencing analysis
Source: BMC Med Genomics. 2024 Oct 4;17:244. doi: 10.1186/s12920-024-02017-z (PMC11451007; doi:10.1186/s12920-024-02017-z)
Supplement: Supplementary file 1 — Supplementary Material 1. [file 12920_2024_2017_MOESM1_ESM.docx]

**Supplementary Materials for**

***CLN3* transcript complexity revealed by long-read RNA sequencing analysis**

Hao-Yu Zhang, Christopher Minnis, Emil Gustavsson, Mina Ryten & Sara E Mole


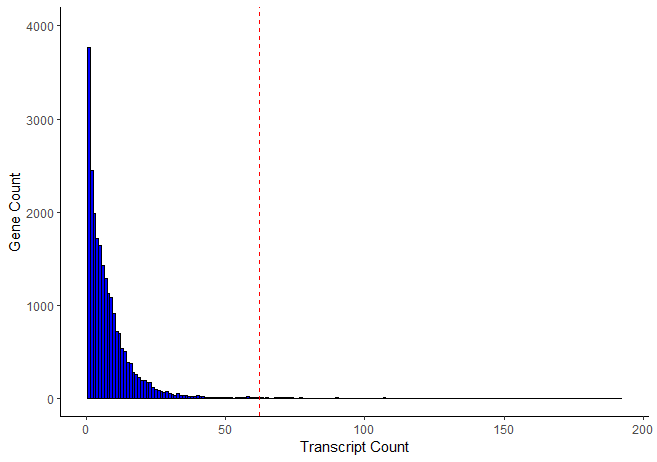


**Figure S1. Distribution of the number of transcripts per gene in Ensembl 110.**

This plot shows the distribution of the number of transcripts per gene in Ensembl 110. The red dashed line marks the position of *CLN3*.

**
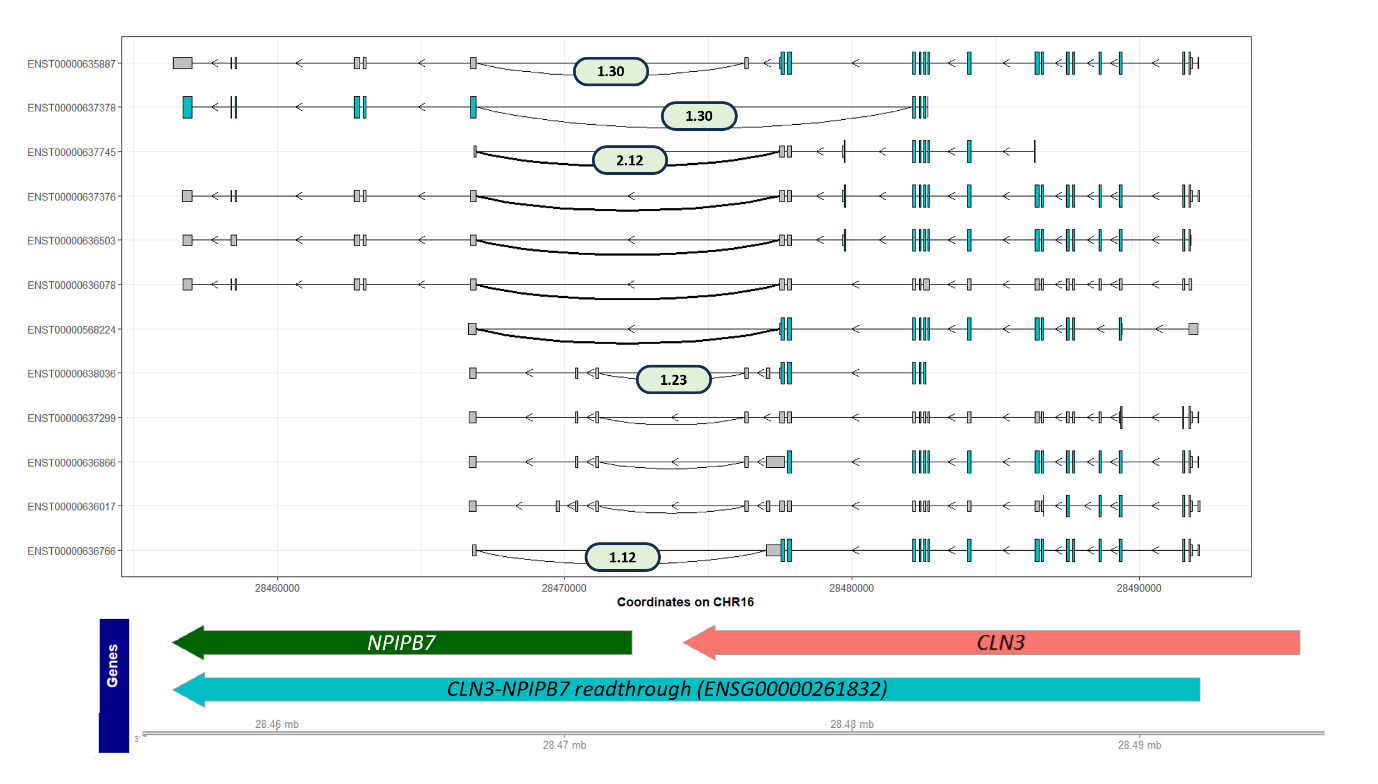
**

**Figure S2. Summary of exon-exon junctions specific to the readthrough gene ENSG00000261832 from GTEx V8.** All five exon-exon junctions with splice donor sites in *CLN3* locus and splice acceptor sites in *NPIPB7* locus are extracted from GTEx V8 exon-exon junction read counts data. All 12 transcripts containing these junctions are plotted, with taller coloured boxes showing the open reading frames (ORFs) and shorter grey boxes showing the untranslated regions (UTRs). Note, these transcripts are present on the antisense strand, so read right to left. These five junctions are shown in curved lines connecting the donor and acceptor sites from “Junction 1” (top) to “Junction 5” (bottom). For each junction, the average read count among all tissue donors and tissue types is shown in boxes above the junction. All five exon-exon junctions are detected in multiple tissues, supporting their existence. Amongst all five junctions, “junction 3” (chr16:28466903-28477463:-) has the highest detection rate at 95.3% (364 tissue donors out of 372) in brain samples and the highest average read counts of 2.12 across all tissues (**Table S1**). “Junction 2” (chr16:28466903-28482104), which is specific to the transcript (ENST00000637378) with an open reading frame containing coding sequences from both CLN3 and NPIPB7, is detected in 63.2% of brain tissue donors (**Table S1**). The gene locus of *CLN3, NPIPB7* and the *CLN3-NPIPB7* readthrough gene are shown at bottom.


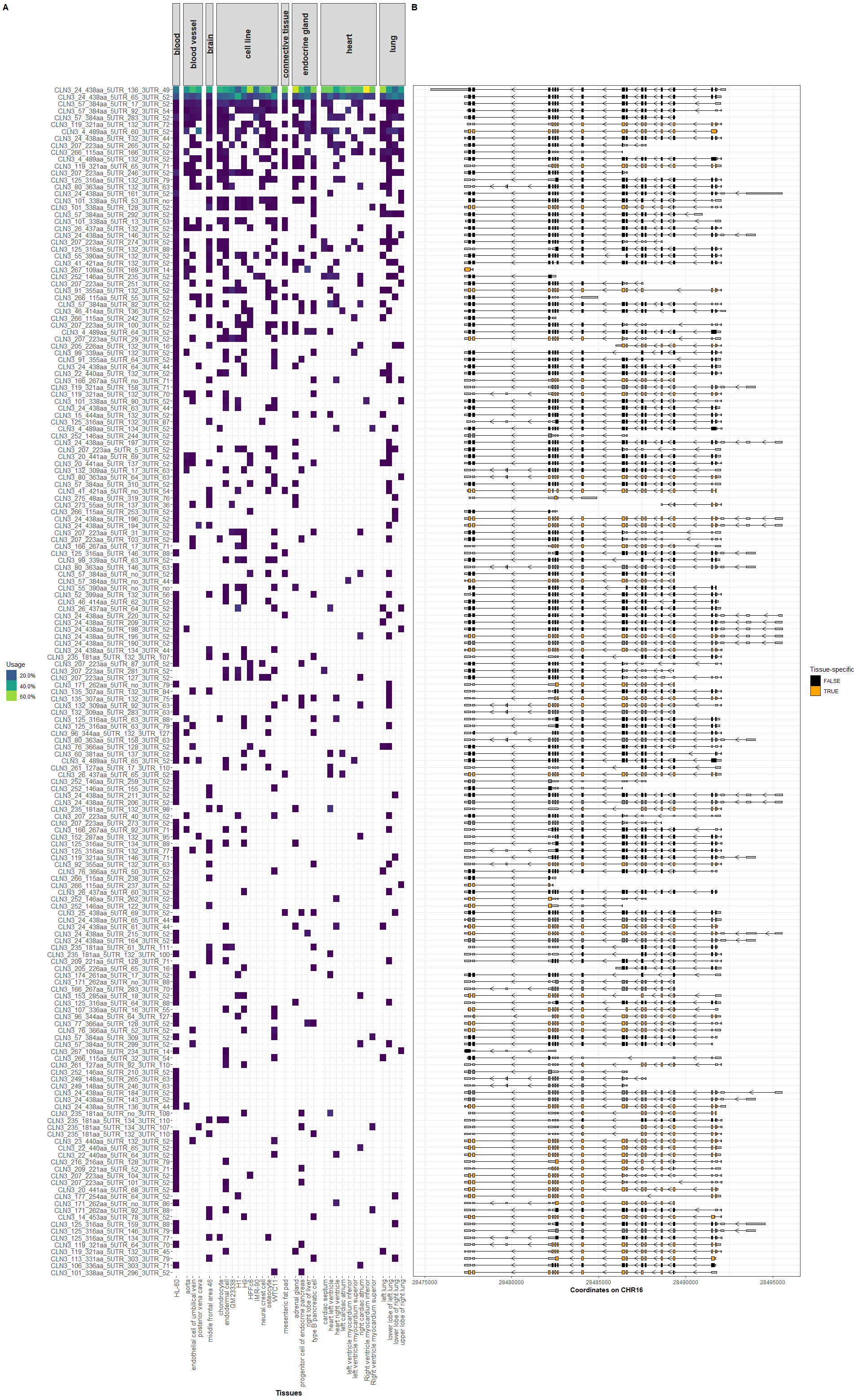


**Figure S3. Usage and tissue-specificity for all 172 valid *CLN3* transcripts.**

Usage of valid transcripts across tissue types is summarised and plotted using heat maps with yellow showing high usage and dark blue showing low usage. Structures of all 172 valid transcripts are plotted, with open reading frames (ORFs) coloured by tissue-specificity. Orange shows tissue-specific transcripts and black shows non-tissue-specific transcripts. Untranslated regions (UTRs) are represented by shorter grey boxes.
